# Quantitative MR Imaging of the Breast

**DOI:** 10.2463/mrms.rev.2025-0085

**Published:** 2025-10-29

**Authors:** Masako Kataoka, Maya Honda, Mami Iima, Takashi Ueguchi, Megumi Matsuda, Takatoshi Aoki, Eun Sook Ko

**Affiliations:** 1 Preemptive Medicine and Lifestyle-related Disease Research Center, Kyoto University Hospital, Kyoto, Kyoto, Japan; 2 Department of Fundamental Development for Advanced Low Invasive Diagnostic Imaging, Nagoya University Graduate School of Medicine, Nagoya, Aichi, Japan; 3 Center for Information and Neural Networks, Advanced ICT Research Institute, National Institute of Information and Communications Technology, Suita, Osaka, Japan; 4 Department of Radiology, Ehime University Graduate School of Medicine, Toon, Ehime, Japan; 5 Department of Radiology, University of Occupational and Environmental Health, Kitakyushu, Fukuoka, Japan; 6 Department of Radiology, Samsung Medical Center, Sungkyunkwan University School of Medicine, Seoul, Korea

**Keywords:** breast neoplasms, diffusion weighted imaging, dynamic contrast enhanced magnetic resonance imaging, multiparametric magnetic resonance imaging

## Abstract

Breast MRI has become essential for diagnosing and managing breast diseases. MRI interpretation has traditionally relied on subjective assessment according to Breast Imaging-Reporting and Data System, but recent advances emphasize the value of quantitative MRI data as objective imaging biomarkers. Techniques such as dynamic contrast-enhanced MRI (DCE-MRI), including ultrafast DCE-MRI and diffusion-weighted imaging are the most established for quantitative analysis, yet their routine application is limited by a lack of standardization in imaging protocols. Recent efforts focus on improving reproducibility, with studies showing that significant changes in the apparent diffusion coefficient can reliably indicate biological alterations in breast lesions. Emerging quantitative MRI approaches including synthetic MRI and fat imaging show promise in lesion characterization and treatment response assessment but require further validation and harmonization before widespread clinical use. For methodological aspect, radiomics has demonstrated strong diagnostic and predictive capabilities in breast MRI research. Quantitative approach for background parenchymal enhancement indicated its association with future breast cancer incidence or recurrence. Continued efforts to standardize and validate quantitative MRI parameters are crucial to fully integrating these tools into routine breast imaging practice.

## Introduction

Breast MRI is now becoming the indispensable tool for diagnosis and the management of breast disease. Breast MRI is used as a tool for problem-solving after the inconclusive results on mammography or ultrasonography, for preoperative assessment, for screening of women with increased risk of breast cancer, and for evaluation of treatment response.^1^ The MRI section of the American College of Radiology Breast Imaging-Reporting and Data System (BI-RADS) is widely used as a guide to describe breast MRI.^2^ In BI-RADS, lesion evaluation largely depends on the reader’s assessment. However, recent studies have shown that various quantitative data can aid in the diagnosis of lesions, the evaluation of therapeutic efficacy, and the prediction of future breast cancer risk. The advantage of quantitative MRI includes reproducibility, objectiveness, and relative independence of image acquisition. Quantitative data derived from MRI is regarded as imaging biomarkers.^3^ This review article explores the benefits of quantitative evaluation, starting from kinetic analysis in contrast-enhanced MRI and diffusion-weighted imaging (DWI), including phantom studies to standardize DWI. New quantitative data derived from synthetic MRI (SyMRI) and fat imaging are also discussed for their role in the diagnosis and the evaluation of breast lesions.

## Contrast-enhanced MRI

Standard breast MRI relies on dynamic contrast-enhanced (DCE) techniques. In addition to morphological information, the perfusion aspects of DCE have been incorporated into the BI-RADS. Information of enhancement at a minimum of 3 time points is required to draw a time-intensity curve and perform qualitative analysis,^2^ i.e. slow, medium or fast for the initial phase and persistent, plateau or washout for the delayed phase. In general, hypervascular lesions demonstrate a fast/washout pattern, indicating malignancy.

**Fig. 1:**
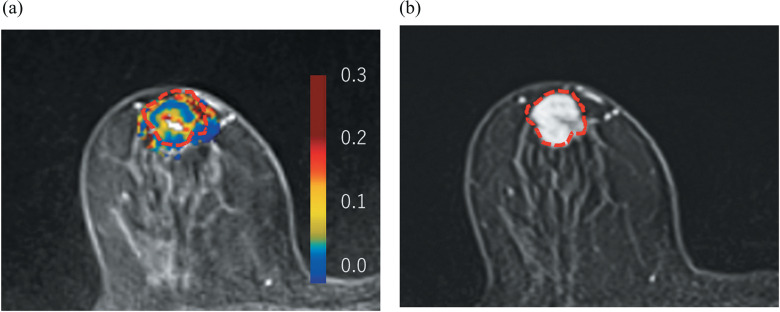
A representative Ktrans map of a woman in her 60s who underwent neoadjuvant chemotherapy for her invasive breast carcinoma in her right breast. (**a**) The Ktrans color map overlayed on ultrafast dynamic contrast enhanced MRI demonstrated intratumoral heterogeneous perfusion, with a mixture of high Ktrans (yellow & red) and low Ktrans (blue) within the lesion (indicated by the red-dotted line). Median of Ktrans was 0.18/min. (**b**) Initial phase of dynamic contrast enhanced MRI of the same lesion demonstrated more homogeneous enhancement pattern.

Efforts have been made to quantify the vascular/perfusion aspect of breast lesions. The two major approaches are “a fully quantitative pharmacokinetic analysis for tissue perfusion and capillary permeability, including the Tofts model” and “a semi-quantitative analysis based on the time-intensity curve in DCE, including the wash-in slope or maximum slope.” The most well-known quantitative approach is the Tofts model.^4^^–^^6^ The model assumes that the contrast agent is exchangeable between the blood plasma and interstitial fluid compartments of the tissue. The Tofts model uses the concentrations of the contrast agent in the tissue over time to estimate various parameters and provides key parameters for characterizing tissue perfusion and permeability. Several parameters derived from Tofts model have been investigated for their clinical utility. For example, the transfer constant Ktrans, which represents the rate of transfer of the contrast agent from the blood plasma into the extravascular extracellular space (EES), is higher in malignant lesions than in benign lesions and can therefore be used to diagnose breast cancer.^7^ Ktrans is also associated with aggressive histopathological findings of breast cancer (Fig. 1). Other key parameters from Tofts model include fractional volume (ve), defined as the fractional volume of EES relative to the tissue and interstitial volumes. It is the space in which contrast agent accumulates after crossing the endothelial barrier. Another important parameter is the rate constant (kep), the rate of reflux of contrast agent from the EES to plasma. Both of them have also been investigated as imaging markers for the diagnosis and characterization of breast cancer.^8^^,^^9^

The impact of scan duration on pharmacokinetic parameters derived from the Tofts model in breast DCE-MRI was investigated. A scan duration of at least 5 mins post-contrast injection is sufficient for reliable estimation of parameters while shorter scan durations (e.g., 1–2 mins) led to significant deviations in parameter values, especially in malignant lesions.^10^ The study comparing high–temporal-resolution (11 seconds per phase) and low–temporal-resolution (60 seconds per phase) DCE-MRI in assessing BI-RADS category 4 breast lesions found the high–temporal-resolution protocol provided better discrimination between benign and malignant lesions, emphasizing the importance of temporal resolution in pharmacokinetic modeling.^11^ However, obtaining sufficiently high–temporal-resolution images for over 5 mins is challenging in the busy clinical practice.

Ultrafast DCE (UF-DCE) MRI of the breast, proposed in 2014 by Mann et al.,^12^ focuses on the very early phase after administration of a contrast agent. Several accelerated scanning techniques (view-sharing, compressed sensing, etc.) contributed to the development of UF-DCE techniques. These techniques have been used to capture kinetic data within seconds of contrast agent administration, while maintaining reasonable spatial resolution for morphological assessment. UF-DCE MRI provides additional parameters from the early contrast uptake phase, improving or matching the diagnostic performance compared with standard DCE analysis. Kinetic parameters obtained from UF-DCE MRI include maximum slope (MS), representing the steepness of the upslope of the time-intensity curve, time to enhancement (TTE) and bolus arrival time (BAT), which indicates when the breast lesion starts to enhance. The MS of breast cancer is larger than that of benign lesions, while TTE and BAT of breast cancer are shorter than those of benign lesions.^7^^,^^13^^,^^14^ UF-DCE MRI also allows the detailed depiction of tumor-related vessels, which is developed due to increased vascularity of breast lesions.^15^^–^^17^ Nevertheless, at present, the clinical application of these parameters is limited by the lack of uniformity in acquisition methods. Standardization of acquisition parameters is necessary for the widespread use of ultrafast MRI. Further discussions are found in the paper by Yamaguchi et al.^18^

**Fig. 2:**
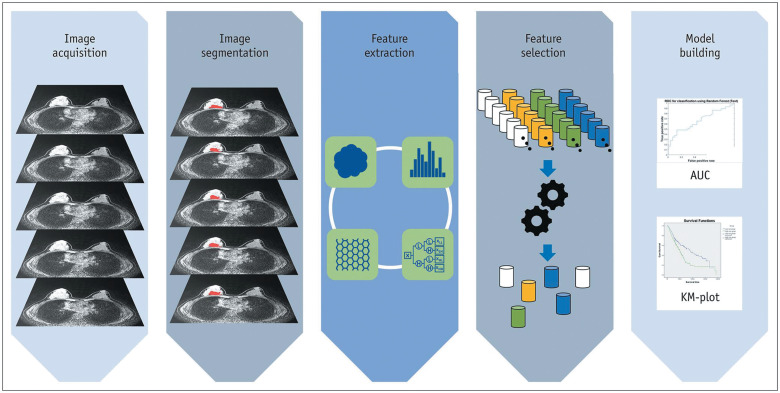
Overview of the steps in radiomics studies. Reprinted from the reference Lee et al.^87^ AUC, area under curve; KM, Kaplan–Meier.

Along with the technical aspects, methodological advancement needs to be mentioned. One is the application of radiomics in breast MRI. Radiomics uses advanced computational techniques to extract high-throughput quantitative features from medical images, providing insights beyond what is visible to the human eye.^19^^,^^20^ Key steps include image acquisition, ROI specification, feature extraction, feature selection, and model building (Fig. 2). Textural features, particularly those based on the gray-level co-occurrence matrix, are critical in capturing tumor heterogeneity.^21^ Feature selection then identifies the most relevant features for the desired response variable.^22^^,^^23^ The final step involves building models using these selected features for classification or regression tasks. For distinguishing between benign and malignant breast lesions,^24^^–^^26^ combining radiomic features from DCE-MRI with pharmacokinetic parameters has shown to improve tumor discrimination. Radiomics also shows promise in predicting the response to neoadjuvant chemotherapy.^27^^–^^29^

Another emerging area of quantitative approach in contrast-enhanced MRI is the evaluation of background parenchymal enhancement (BPE). The higher level of BPE at breast MRI is potentially associated with the presence and the risk of developing breast cancer. However, results have been in conflicting. A metanalysis found the association of higher BPE and breast cancer only among high-risk women, while not among average-risk women.^30^ In this analysis, most of the studies used 4-tier qualitative analysis of BPE based on BI-RADS. Recent studies use quantitative measurement of BPE to identify association of higher BPE with future breast cancer incidence^31^^,^^32^ or recurrence.^33^^–^^37^ Again, intra-individual comparison of BPE measurement methods demonstrated variation among methods which requires standardization. The authors concluded that intensity-based methods correlate more closely with radiologists’ ratings than volume-based methods.^38^

## Breast DWI: Merit for ADC and Quantitative Values

DWI has become an essential component of breast MRI protocols, offering valuable insights into tissue microstructure without the need for contrast agents. The apparent diffusion coefficient (ADC) derived from conventional DWI has shown significant promise in characterizing breast lesions, monitoring treatment response, and correlating with important pathological markers.

**Fig. 3:**
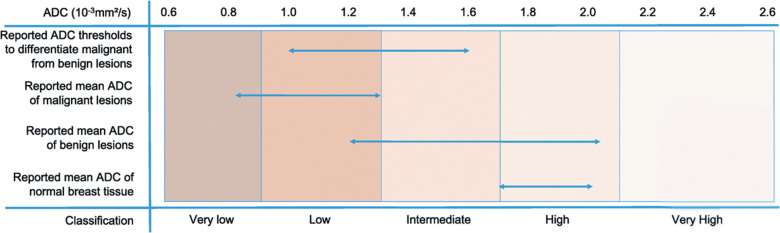
ADC thresholds and value ranges for malignant, benign, and normal tissue. In this graph, the lower horizontal arrows show the range of reported mean ADC values for normal breast tissue, benign, and malignant lesions. The top arrow shows the range of suggested thresholds to differentiate between benign and malignant lesions. Note that this graph simply lists ranges as taken from the original tables and no data pooling was performed. The color bars correspond to the diffusion levels that were defined and agreed upon by the working group in order to standardize the description of the diffusion values. Adopted from reference Baltzer et al.^39^ADC, apparent diffusion coefficient.

The diagnostic value of ADC in differentiating malignant from benign breast lesions has been well-established through numerous studies and meta-analyses. Malignant lesions typically exhibit lower ADC values compared to benign lesions and normal breast tissue, reflecting their increased cellularity and restricted water diffusion. Meta-analyses have reported ADC ranges of 0.8–1.3 × 10^−3^ mm^2^/s for malignant lesions, 1.2–2.0 × 10^−3^ mm^2^/s for benign lesions, and 1.7–2.0 × 10^−3^ mm^2^/s for normal breast tissue.^39^ This clear distinction in ADC values has led to the development of threshold-based classification methods, potentially reducing unnecessary biopsies and improving diagnostic accuracy (Fig. 3).^40^^,^^41^

In recent years, advanced non-Gaussian diffusion models have emerged, extending beyond the limitations of standard ADC measurements. These include techniques such as diffusion kurtosis imaging,^42^ which captures the non-Gaussian behavior of water diffusion in complex tissues, and intravoxel incoherent motion (IVIM), which separates the contributions of tissue perfusion and diffusion.^43^ These advanced methods provide additional quantitative parameters that may offer more detailed information about tissue microstructure and heterogeneity in breast cancer. Several studies have found that low diffusion coefficient (D) values from IVIM suggest malignancy and are associated with aggressive features of breast cancer and lymph node metastasis. K values from diffusion kurtosis imaging could be used to prospect prognosis in breast cancer patients and to analyze microstructural changes in breast cancer.^44^

## The Role of Phantom Studies

While the clinical potential of ADC and other quantitative DWI measures is evident, their widespread implementation relies heavily on ensuring reproducibility and standardization across different scanners and institutions. This is where phantom studies play a crucial role. Phantoms are specially designed objects that mimic specific properties of human tissue and provide known reference standards for imaging parameters. In the context of breast DWI, phantoms are essential for assessing and maintaining the accuracy of ADC measurements, evaluating the performance of different acquisition protocols, and facilitating quality control in multi-center trials.

One of the most widely used phantoms for breast DWI is the ice-water phantom, which has been employed in several multi-center trials, including American College of Radiology Imaging Network (ACRIN) 6698 and ACRIN 6702.^45^ It consists of a central thin-walled plastic tube filled with water, surrounded by an ice-water bath. This simple yet effective phantom provides a known diffusion coefficient of water at 0°C (approximately 1.1 × 10^−3^ mm^2^/s), which serves as an absolute reference for ADC measurements.^45^ The ice-water phantom allows for the assessment of ADC bias, spatial uniformity, and the impact of gradient non-linearity on ADC measurements.

Another important class of phantoms for breast DWI are those based on polyvinylpyrrolidone (PVP) solutions. PVP phantoms offer the advantage of providing a range of ADC values that span those typically observed in breast tissues. The National Institute of Standards and Technology (NIST), in collaboration with the Quantitative Imaging Biomarkers Alliance (QIBA), has developed a PVP-based phantom that includes vials with different PVP concentrations, allowing for the assessment of ADC linearity and bias across a clinically relevant range of diffusion coefficients.^46^ This phantom also incorporates features for evaluating spatial distortion, resolution, and other imaging parameters critical for breast DWI.

**Fig. 4:**
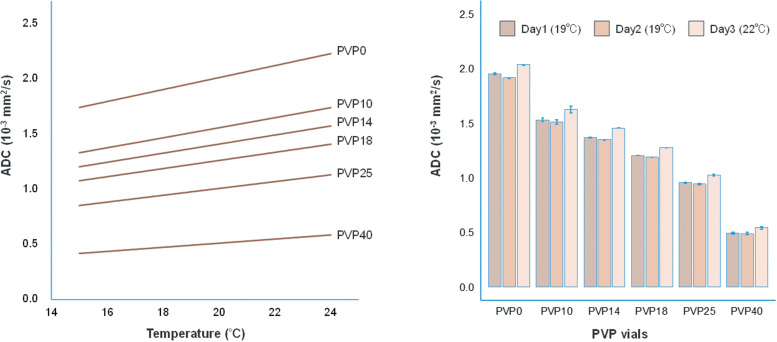
The left graph shows the correlation between temperature and ADC value for each PVP vial, derived from NIST reference data, demonstrating that even slight differences in phantom temperature can affect ADC measurements. The right graph presents ADC values measured over 3 non-consecutive days. While Days 1 and 2 had nearly identical temperatures, Day 3 experienced a temperature difference of approximately 3°C. The ADC measurements on Day 3 exhibit a notable increase, complicating the evaluation of reproducibility. Note that the numbers following ‘PVP’ indicate the concentration of polyvinylpyrrolidone in each vial (e.g., PVP40 represents vials with 40% PVP concentration).ADC, apparent diffusion coefficient; NIST, National Institute of Standards and Technology; PVP, polyvinylpyrrolidone.

A commercially available breast DWI phantom (CaliberMRI, Boulder, CO, USA) based on the NIST design has been developed, featuring bilateral inserts with PVP vials and geometric targets for assessing spatial accuracy, as well as features for evaluating spatial distortion, resolution, and other imaging parameters critical for breast DWI.^47^ It also includes fat-mimicking components for a more realistic representation of breast tissue. This phantom includes an MR-visible liquid crystal thermometer, enabling precise temperature measurement during scanning. As illustrated in Fig. 4, temperature control is crucial for phantom studies, as the diffusion coefficient of water and PVP solutions is temperature-dependent. The ability to accurately measure phantom temperature allows for more precise ADC quantification and system performance evaluation. Phantom studies have been instrumental in identifying and addressing sources of variability in ADC measurements across different scanner platforms and field strengths (Fig. 4).

The use of phantoms in breast DWI extends beyond technical validation to play a crucial role in multi-center clinical trials. For example, in the ACRIN 6698 trial, which evaluated ADC as a predictor of treatment response in neoadjuvant chemotherapy, phantom scans were required for site qualification and ongoing quality control.^48^ This approach ensured consistent scanner performance across sites and time points, enhancing the reliability of the trial’s ADC measurements and conclusions. Although not common as ADC, IVIM may be used in future multi-center clinical trials, as the reproducibility of IVIM parameters across different sites and vendors has been demonstrated in a phantom study.^49^

Phantoms also facilitate the development and validation of new image processing techniques for breast DWI. For instance, denoising algorithms, which aim to improve image quality and ADC precision, can be rigorously tested on phantoms with known properties before being applied to clinical data. Similarly, methods for correcting geometric distortions in DWI, a common issue in breast imaging due to susceptibility effects, can be optimized using phantoms with well-defined geometric features.^50^^,^^51^

While phantom studies have significantly advanced the quantitative capabilities of breast DWI, challenges remain. One limitation of current phantoms is their inability to fully replicate the complex microstructure and heterogeneity of breast tissues. Efforts are underway to develop more sophisticated phantoms that better mimic the diffusion properties of different breast tissue types and lesions. Additionally, the development of anthropomorphic breast phantoms that simulate realistic breast shapes and include both adipose and fibroglandular tissue equivalents is an active area of research. These advanced phantoms could provide more clinically relevant test objects for optimizing breast DWI protocols and analysis methods.

The choice of fat suppression method also impacts ADC values and image quality in breast DWI, with short-tau inversion recovery (STIR) and spectral adiabatic inversion recovery (SPAIR) being two prominent techniques.^52^ STIR, while effective in suppressing fat signals, is not specific to fat and can suppress signals from other tissue components with similar T1 values, potentially leading to biased ADC measurements. This non-specificity can result in over- or underestimation of ADC values, particularly in heterogeneous lesions. In contrast, SPAIR offers higher specificity to fat signals, providing better SNR, improved contrast-to-noise ratio, and more accurate ADC measurements. Studies have shown that DWI-SPAIR provides higher sensitivity in lesion discrimination compared to DWI-STIR. However, SPAIR’s effectiveness depends on good B0 field homogeneity, and imperfect shimming can lead to incomplete fat suppression. Despite this limitation, SPAIR is generally recommended by international bodies such as the European Society of Breast Imaging^39^ and QIBA for breast DWI due to its overall superior performance in maintaining the integrity of water-based diffusion measurements and providing more reliable quantitative assessments.

In addition to acquisition methods, the ADC value is greatly influenced by the placement of the ROI in breast tumors *in vivo*.^53^

In summary, ADC and quantitative values from breast DWI represent powerful tools in breast imaging. As standardization efforts progress and advanced techniques are refined, the role of these quantitative measures in breast cancer diagnosis, characterization, and treatment monitoring is likely to expand. Continued research and collaboration among radiologists, physicists, and oncologists will be crucial in fully realizing the potential of these imaging biomarkers and translating them into improved patient care and outcomes in breast cancer management.

In the clinical context, radiomics helps in improving performance of DWI. Dong et al.^54^ reported that radiomic features from DWI were more strongly correlated with sentinel lymph node metastases than traditional ADC mapping. DWI is often a part of multi-parametric MRI, in which radiomics showed excellent results as high as area under curve (AUC) of 0.89 when applied to multi-parametric MRI.^55^^,^^56^

## Usefulness of SyMRI for Diagnose of Breast Lesions

**Fig. 5:**
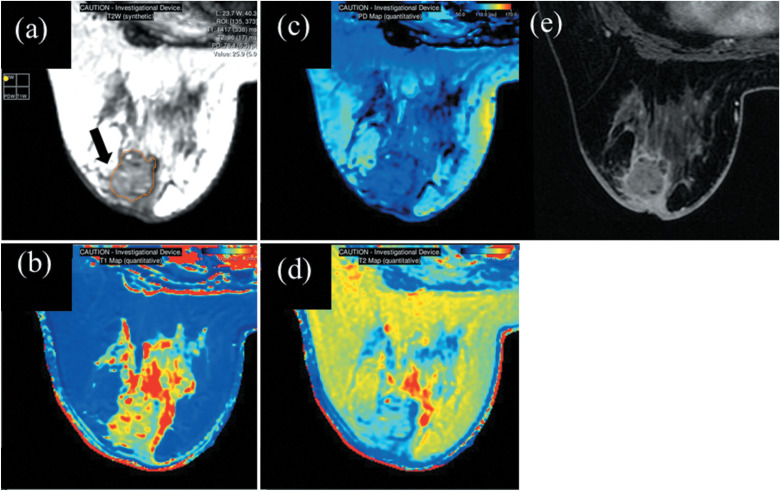
A woman in her 40s with HER-2 enriched breast cancer (cT4bN2M0 cStageIIIB). Pre contrast SyMRI (synthetic T2-weighted image: **a**, T1 map: **b**, PD map: **c**, T2 map: **d**) and DCE-MRI (**e**) show a lobulated mass enhancement with irregular shape and rim enhancement in the left breast (arrow). A ROI to obtain quantitative values in SyMRI was manually drawn to encompass as much of the abnormality as possible and stay within the border of the breast lesion using DCE-MRI as a reference and placed in the synthetic T2-weighted image. The T1, T2, PD, SD of T1, SD of T2, and SD of PD of the lesion obtained from SyMRI using ROI are1417 ms, 96 ms, 78.3 pu, 338, 17, and 6.3, respectively. DCE, dynamic contrast-enhanced; PD, proton-density; SD, standard deviation; SyMRI, synthetic MRI.

Beyond DWI, quantitative assessment of other non-contrast MRI sequences also contributes to the diagnostic evaluation of breast cancer. T1 and T2 mapping—quantitative techniques that measure the longitudinal (T1) and transverse (T2) relaxation times of tissues—have emerged as promising non-contrast imaging biomarkers. The recently proposed SyMRI is a compilation pulse sequence that uses a multi-dynamic multi-echo (MDME) sequence.^57^^,^^58^ SyMRI can perform T1-weighted, T2-weighted, proton-density (PD)–weighted, and inversion recovery imaging and measure absolute values for tissue properties such as T1, T2 and PD values in one single scan (Fig. 5).^59^^,^^60^ These quantitative values demonstrated good accuracy and reproducibility, even across instruments from different vendors.^59^^,^^61^^–^^64^ SyMRI can reduce the scanning time. It takes just 3–5 mins to scan breast SyMRI, which increases clinical utility. Notably, SyMRI enables the acquisition of T1-weighted, T2-weighted, and PD-weighted images within a scan time comparable to that required for conventional T1- and T2-weighted imaging alone. Recently, the clinical utility of these quantitative values obtained from SyMRI for breast lesions have been reported.^65^^–^^67^

The quantitative values obtained from SyMRI are useful in differentiating malignant and benign breast lesions. The difference in cell composition and microstructure affects the magnetization characteristics of the tissue, which might be characterized by the T1 and T2 values.^68^ The differences of T1 and T2 values were related to the content of free water; the higher free water content, the longer the T1 and T2 relaxation times. In particular, T2 value has a better diagnostic performance than other quantitative values.^69^^,^^70^ In malignant lesions, continuous cell proliferation and the release of necrotic material lead to a reduction of the extra cellular space,^71^ which explains decrease in tissue free water content and shorter T2 value among malignant lesions. T1 and T2 values obtained from SyMRI (including pre contrast T1^72^ and T2 values^68^ and post contrast T2 value)^65^ are useful in differentiating malignant and benign breast lesions.

The PD value reflects the total amount of free water and bound water, which is different from T2 value.^65^ The total amount of water molecular content and distribution of water molecules in the breast lesion differ between benign and malignant lesions, which helps in differential diagnosis. Moreover, combining SyMRI with DCE-MRI and DWI can further improve the diagnostic performance, especially for BI-RADS category 4 lesions.^68^^,^^72^

Quantitative values obtained from SyMRI are useful in predicting subtype^73^ and response to neoadjuvant chemotherapy^74^^,^^75^ in breast cancer. However, there are variations in how to measure quantitative values obtained from SyMRI including size and shape of the ROI. Standardized method of ROI placement is desirable to ensure the reproducibility of these quantitative values. Moreover, the MDME sequences are highly optimized for brain tissue while noise, partial volume effect and B1 effects could cause systematic errors and compromise the accuracy of the calculated relaxation times.^76^ Therefore, further refinement of the method optimized for breast^77^ are needed for accurate quantification using SyMRI.

## Imaging of the Fat: Peritumoral Fat and Tumor Aggressiveness

The tumor microenvironment plays a key role in breast cancer development, and growing attention is being paid to the involvement of peritumoral breast adipose tissue in association with tumor aggressiveness and lymph node metastasis. Breast cancer cells secrete soluble factors leading to adipocyte “dedifferentiation” and these adipocytes secrete soluble factors (interleukin-6 [IL-6] and hepatocyte growth factor [HGF]), exosomes, and extracellular matrix components (matrix metalloproteinase-11 [MMP-11] and collagen IV), and then promote tumor cell invasiveness.^78^^,^^79^ Histologically, the adipocytes undergo morphologic changes, first marked by a decrease in adipocyte size and lipid content, then by acquiring a fibroblast-like morphology.^80^ In addition, the cytokine release from the peritumoral adipocytes leads to peritumoral edema.^78^ These histological changes in the microenvironment of the breast result in decreased peritumoral fat proportion, which may predict tumor aggressiveness and lymph node metastasis.

As a tool to noninvasively assess the fat-water contents, chemical shift–based water-fat separation techniques employ an uncomplicated signal model that adopts a single resonant frequency for both water and fat. Nevertheless, T2^*^ decay is a confounding factor to estimate fat content correctly in areas of irregular shape and abrupt changes between soft tissue and air, such as breast. Iterative decomposition of water and fat with echo asymmetry and least-squares estimation (IDEAL) imaging can steadily separate fat and water by using asymmetric echo times and the multi-point Dixon method.^81^ Calculation of the quantity of magnetic inhomogeneity in each pixel from data using the least-squares method was employed to generate the field map of IDEAL, and phase shift is corrected in each pixel by using this field map and create images with accurately divided water and fat. Regarding fat fraction measurement, excellent correlation between T2^*^-corrected IDEAL method (known as IDEAL-IQ) and MR spectroscopy (the most accurate method) is reported.^82^

**Fig. 6:**
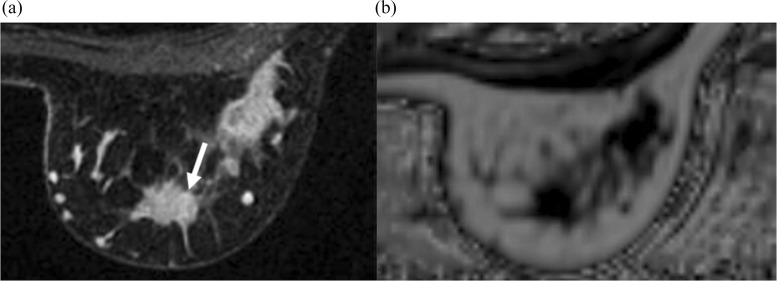
Representative case of breast carcinoma with axillary lymph node metastasis and the high percentage of MIB-1 index. Fat-suppressed post-contrast T1-weighted image (**a**) shows the enhancing mass (arrow) in the left breast, and the corresponding average peritumoral FFt (76.7) on the fat fraction map (**b**) and the pTFR (0.84) are low. Reprinted from reference Hisanaga et al.^83^ FFt, fat fraction values; pFTR, peritumoral fat ratio.

Since IDEAL is simple to perform in a short time (about 30 seconds) without an extrinsic contrast agent, significance of peritumoral fat contents in the clinical context has been explored. For example, peritumoral fat content using IDEAL-IQ is associated with the lymph node metastasis and the MIB-1 index,^83^ and recurrence free survival, but not with peritumoral edema grades on T2-weighted images and ADC generated by DWI (Fig. 6).^84^ Although preliminary, the quantification of the peritumoral fat content identified by using IDEAL-IQ may be a biomarker of prognosis among breast carcinoma patients.

## Potential of Quantitative MRI as Imaging Biomarkers of Breast Cancer

Multiple quantitative MRI techniques have been applied in breast imaging to enhance diagnostic and prognostic accuracy. Among them, pharmacokinetic modeling with DCE-MRI and DWI are the most widely utilized sequences. However, their application as quantitative biomarkers remains limited due to incomplete standardization across imaging protocols. Considerable efforts have been directed toward improving repeatability and reproducibility, including the use of dedicated phantoms to calibrate measurements.^45^^,^^47^ Notably, changes in the mean ADC exceeding 8%–15% in breast lesions have been reported to reflect true biological changes with 95% confidence.^85^

UF-DCE MRI is an emerging technique that introduces several novel kinetic parameters with potential clinical relevance. While these parameters show promise in lesion characterization and treatment response prediction, their utility as quantitative biomarkers is still in the preliminary stages due to limited progress in protocol harmonization and validation.^7^^,^^13^

Similarly, SyMRI and IDEAL represent newer quantitative approaches, which leverage the intrinsic tissue-characterizing capabilities of MRI. Their encouraging early results suggest potential as novel imaging biomarkers, although further validation is needed.

Radiomics is also gaining traction in breast MRI research owing to its strong diagnostic and predictive performance. Nonetheless, its clinical translation is challenged by significant methodological variability, which limits their reliability and reproducibility in clinical settings.^86^

## Conclusion

This review summarizes current quantitative MRI techniques and parameters relevant to breast imaging. Among these, DCE-MRI and DWI are relatively well-established and are progressing toward standardization. In contrast, emerging techniques such as UF-DCE, SyMRI, and IDEAL show promise but remain in earlier stages of clinical validation. Continued efforts are essential to advance the quantitative capabilities of breast MRI and facilitate their integration into routine clinical practice.
